# Arthroscopic management of comminuted fracture of the scapular glenoid secondary to electrical shock injury: a case report and literature review

**DOI:** 10.3389/fsurg.2025.1662146

**Published:** 2025-10-03

**Authors:** Zi Zhang, Binyang Meng, Wenhe Li, Qi Wang, Jiangang Cao

**Affiliations:** 1 Department of Sports Injury and Arthroscopy, Tianjin Hospital, Tianjin University, Tianjin, China; 2 Medical School of Tianjin University, Tianjin University, Tianjin, China

**Keywords:** electrical shock injury, glenoid fracture, arthroscopic technique, fracture fixation, case report

## Abstract

Arthroscopic management of scapular glenoid fractures caused by electrical injury represents an innovative approach for complex shoulder trauma involving both osseous and soft tissue damage. This technique uniquely combines the double-pulley system with a 3.0-mm double-suture anchor bridge fixation, allowing for smaller incisions and reduced surgical trauma. We report, for the first time, an arthroscopic case of comminuted anteroinferior glenoid fracture resulting from electrocution. A 53-year-old man presented with left shoulder dysfunction 8 days after electrical injury. CT and MRI revealed a comminuted glenoid fracture, a non-displaced greater tuberosity fracture, and a partial supraspinatus tear. Arthroscopic anchor fixation achieved anatomic reduction of the glenoid fragment without intraoperative complications, while the greater tuberosity fracture and rotator cuff injury were managed conservatively. At 15-month follow-up, the patient was pain-free (VAS score 0) with full shoulder function (Constant score 95, ASES score 94), and CT confirmed satisfactory glenohumeral congruency. This case demonstrates the technical feasibility of arthroscopic treatment for high-energy electrical shoulder trauma, with advantages of minimizing soft tissue disruption and reducing the risk of postoperative stiffness, though further studies are needed to validate long-term outcomes.

## Introduction

In developed countries, electrical injuries account for approximately 3%–5% of all burn cases, whereas in developing countries the incidence is as high as 21%–27% (1–3). Fractures caused by electrocution are rare and usually result from either tetanic muscle contractions or falls secondary to the injury. Although previous reports have described vertebral compression fractures and posterior shoulder dislocations following electroconvulsive therapy or accidental electric shock (4–6), anterior glenoid fractures secondary to electrocution remain exceedingly uncommon.

Tarquinio et al. (7) first reported a case of bilateral scapular fractures after low-voltage electrical injury, attributing to forceful contractions of the shoulder muscles. Subsequent studies by Beswick et al. (8) and Dumas et al. (9) further emphasized the role of intense tetanic contraction in scapular fractures occurring without direct trauma. However, most of these reports focused on isolated scapular body fractures. Accordingly, the commonly recognized injury pattern after electrocution involves posterior muscle contraction leading to posterior shoulder dislocation, posterior glenoid rim fractures, or scapular body fractures (10).

It is noteworthy that the most frequent upper limb injury after electrical trauma is posterior fracture-dislocation of the proximal humerus (6, 11, 12). In contrast, the present patient sustained a rare combination of a comminuted anteroinferior glenoid fracture with a concomitant nondisplaced greater tuberosity fracture. This injury pattern poses diagnostic challenges, for which computed tomography (CT) and magnetic resonance imaging (MRI) are critical in detecting comminuted glenoid fractures and associated soft tissue injuries.

This research reports a case of comminuted anteroinferior glenoid fracture following electrical injury, which to our knowledge represents the first successful arthroscopic management of an electrocution-related glenoid fracture. The concomitant nondisplaced greater tuberosity fracture and partial rotator cuff tear were treated conservatively. This case highlights the complexity of shoulder injuries induced by electrical trauma and introduces a novel minimally invasive surgical option for their management.

## Presentation of case

A 53-year-old man sustained an electrical injury while bending over to touch a generator, with an estimated contact time of 3–5 s. He reported sharp, burning pain in the left upper arm and shoulder, followed by numbness, restricted mobility, local swelling, and tenderness several hours later. He was unable to actively elevate the left arm. No chest pain, palpitations, or trauma from falling were reported. Eight days of post-injury, he presented to our clinic. Physical examination revealed no obvious shoulder deformity but marked tenderness over the coracoid process, greater tuberosity, and bicipital groove. Active/passive ranges of motion were as follows: forward flexion 45°/90°, extension 10°/20°, adduction 10°/20°, abduction 40°/70°, and internal rotation to the lateral thigh. Radial and ulnar pulses were intact, with preserved sensation and muscle strength in all extremities. Electrocardiography, chest radiography, and routine blood tests were within normal limits.

**Figure 1:**
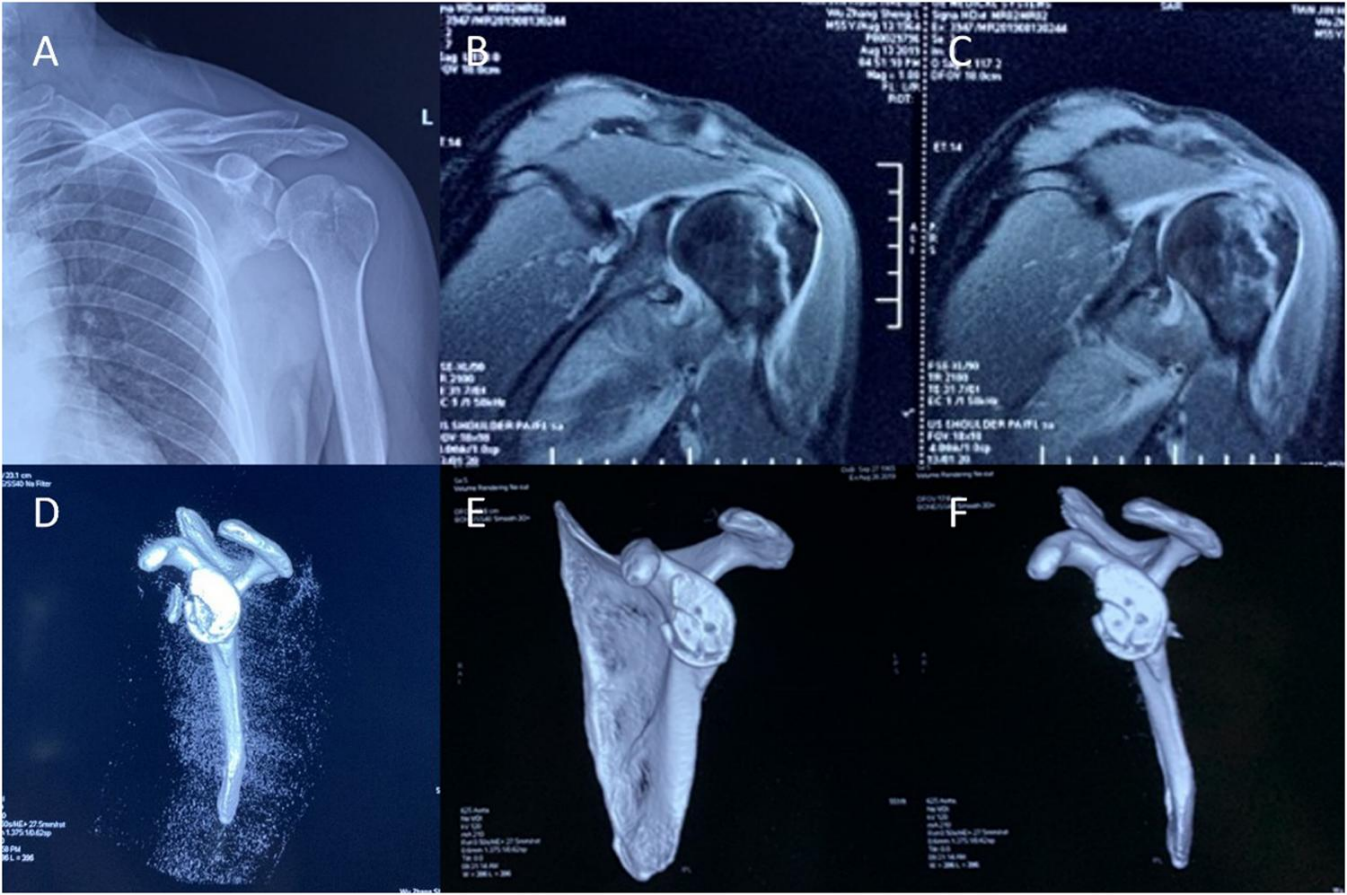
Images of the patient. **(A–D)** Preoperative images. **(E,F)** Postoperative images.

Radiographic and MRI evaluation identified a nondisplaced fracture of the left greater tuberosity (Figure 1A) and a fracture involving the anteroinferior glenoid rim with associated labral involvement (Figures 1B,C). MRI additionally revealed a partial tear of the supraspinatus tendon. CT confirmed the greater tuberosity fracture and demonstrated a comminuted anteroinferior glenoid fracture (Figure 1D). Preoperatively, the patient's pain score was 9 on the Visual Analog Scale (VAS scale), with a Constant-Murley Score (Constant score) of 33 and an American Shoulder and Elbow Surgeons Standardized Shoulder Assessment (ASES score) of 13.

## Surgery procedure

**Figure 2:**
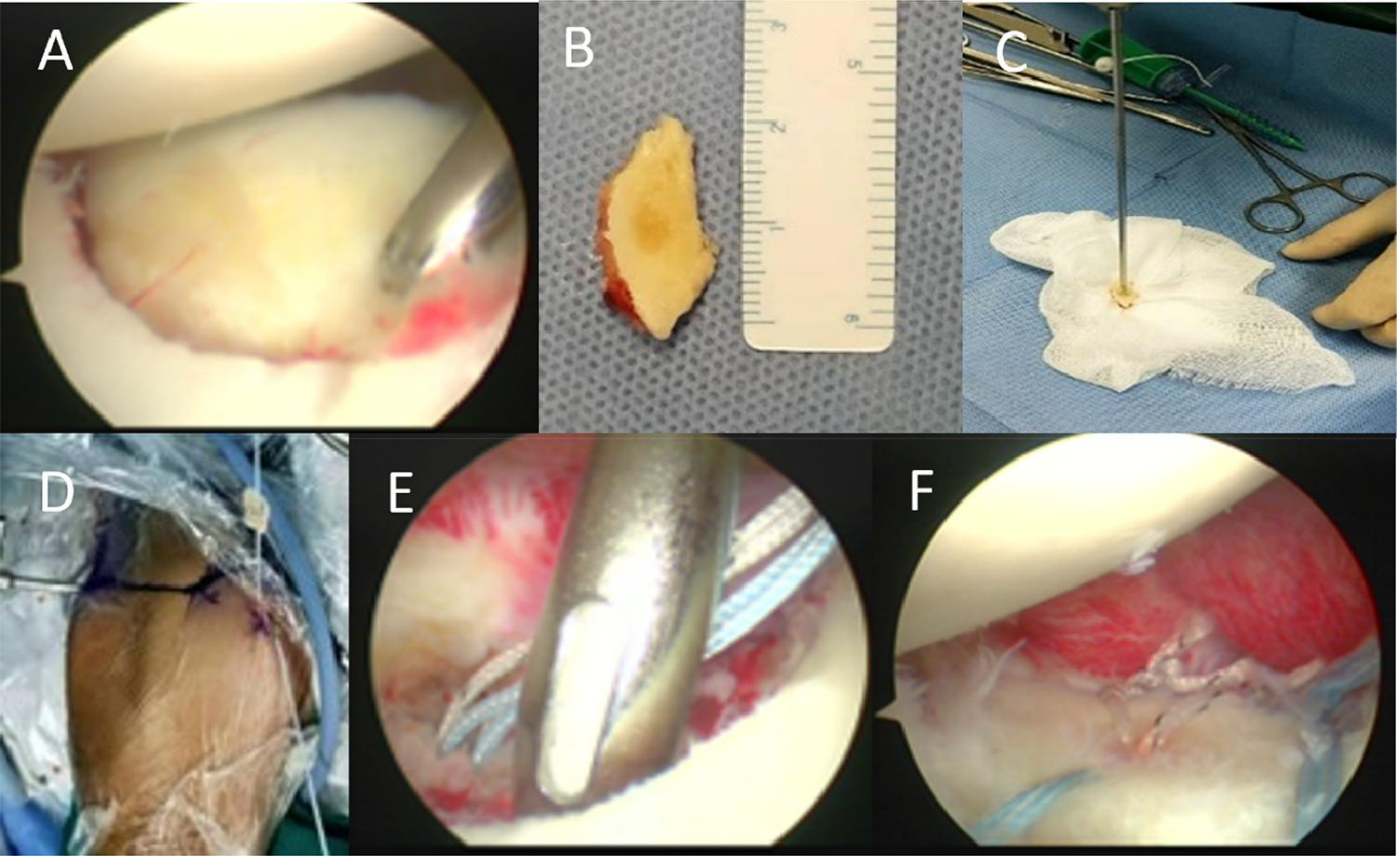
Several major key points in the patient's surgery. **(A)** Compare the size of the bone defect. **(B)** Measure the dimensions of the bone fragment. **(C)** Drill a hole in the center of the bone fragment. **(D)** Reduce the bone fragment into the joint. **(E,F)** Surgical fixation of the bone fragment.

The patient was positioned in the lateral decubitus position with longitudinal traction applied, and preoperative manipulation restored normal range of motion to the left shoulder. Following standard aseptic preparation and draping, anatomical landmarks including the acromion, coracoid process, and acromioclavicular joint were marked. Standard posterior, anterosuperior, and anteroinferior portals were established. Arthroscopic examination identified a displaced anteroinferior glenoid rim fracture, with a free bone fragment displaced anterior to the glenoid cavity. Notably, the labral structure remained intact without tearing (Figure 2A).

After debridement of the fracture site, the free fragment was visualized, preserving its intact labral attachment. Reduction attempts revealed inadequate stability for direct intra-articular fixation. Consequently, the fragment was extracted and measured as 2 cm (length) × 1.5 cm (width) × 0.5 cm (thickness) (Figure 2B). A central drill hole was created in the fragment for subsequent fixation (Figure 2C). Two 3.0-mm double-threaded anchors (Arthrex, Munich, Germany) were implanted at the anteroinferior and central aspects of the glenoid bone bed. Sutures from the central anchor were passed through the pre-drilled hole to reduce the fragment into the joint (Figure 2D). The anteroinferior portion of the fragment was secured using a double pulley technique, while two blue sutures in a suture bridge configuration provided additional stabilization from the anterosuperior and posteroinferior directions (Figures 2E,F). Intraoperative stability testing confirmed rigid fixation.

## Postoperative rehabilitation protocol and follow-up results

Postoperatively, the arm was immobilized with a shoulder sling for 6 weeks to protect the glenohumeral fixation site and facilitate fracture healing. During weeks 1–2, gentle pendulum (Codman) exercises were initiated, avoiding any active shoulder muscle contraction. From weeks 3–6, gradual passive and active-assisted range-of-motion exercises were introduced in forward flexion (scapular plane) and external rotation (with the arm at the side), limited to a comfortable range. Combined abduction–external rotation movements that could stress the anterior repair were strictly avoided. At week 6, the sling was discontinued, and isometric strengthening of the rotator cuff and deltoid muscles was initiated. Between 3 and 6 months, progressive resistance training with elastic bands was performed, targeting internal rotation, external rotation, abduction, and forward flexion. From 6 months onward, advanced strengthening focused on power and endurance.

CT at 6 months demonstrated satisfactory alignment and healing of the glenohumeral fracture (Figures 1E,F). At 15-month follow-up, the patient reported complete resolution of shoulder pain, with restored range of motion. Clinical scores were markedly improved: VAS 0, Constant score 95, and ASES score 94. Imaging confirmed postoperative healing changes in the left glenoid fossa fracture—with good bone repair and a relatively regular shape.

## Discussion

Cases of shoulder fractures caused by electrical injury are rare, with only a few reports available in the literature (Table 1). In the present case, the patient underwent arthroscopic fixation of the glenoid fragment using suture anchors, while the nondisplaced greater tuberosity fracture and partial rotator cuff tear were treated conservatively. At 15 months postoperatively, the shoulder remained stable with full range of motion, excellent functional scores, and imaging confirming well-aligned fracture healing with satisfactory morphology.

**Table 1:** List of publications describing shoulder fractures resulting from electric shock (electrical injury related).

Authors	Year	Injury	Mechanism	Treatment	Follow-up
Tarquinio et al. (7)	1979	Bilateral comminuted scapular fractures (41-year-old)	Electric shock	Immobilizing both upper extremities in slings and early range of motion exercise	The fractures healed without complications and normal function resulted
Beswick et al. (8)	1982	Bilateral scapular fractures (43-year-old)	Electric shock	Conservative management (immobilization, analgesia, and progressive physical therapy)	Six months follow-up (essentially normal shoulder function)
Dumas and Walker (9)	1992	Bilateral comminuted scapular fractures (46-year-old)	Electric shock	Immobilization ice application and analgesics	Two months follow-up (the fractures were healed without loss of motion range of both shoulders)
Kotak et al. (10)	2000	Bilateral extra-articular fractures of the scapulae (51-year-old)	Electric shock	Non-operatively (in slings, with physiotherapy and analgesia)	Three months follow-up (painfree and regained a full range of movements)
Rana and Banerjee (13)	2006	Fracture of the right scapular posterior dislocation (33-year-old)	Electric shock	Broad arm sling and physiotherapy exercises	Three-month follow-up (the scapula was fully healed with no residual tenderness and a return to normal function)
Huang et al. (14)	2010	Posterior comminuted scapular fracture (44-year-old)	Electric shock	Nonoperative immobilization with an arm sling and swathe	Three-month follow-up (pain-free and regained a full range of movement of left shoulder)
Modi et al. (11)	2012	Fracture of the body of the scapula (51-year-old)	Electronic muscle stimulation (EMS)	Broad arm sling	10 weeks follow-up (the scapular was clinically united with no residual tenderness and the range of movement was full in abduction and flexion)
Zbuchea (12)	2015	Comminuted subcapital fracture of the left humerus posterior dislocation (56-year-old)	Electrical injury	Conservative treatment (by immobilization through thoraco-brachial bandage for 30 days)	Discharged the fourth day
Ketenci et al. (6)	2015	Posterior shoulder dislocation (45-year-old)	Electric shock	Closed reduction and orthoses	20 months follow-up (painless and capable of performing all daily activities)

The mechanism of fracture after electrocution is generally attributed to involuntary tetanic muscle contraction or secondary trauma from falls. Notably, the most commonly reported shoulder injuries following electrical trauma are posterior dislocations and posterior fractures. This pattern has been explained by the powerful contraction of muscles such as the infraspinatus, teres minor, and deltoid, which force the humeral head upward and posteriorly against the acromion, resulting in posterior glenoid rim injuries (11, 13, 15). Some authors have suggested that electrocution predominantly leads to posterior dislocation, whereas anterior dislocations are usually trauma related (16). In our case, however, the patient sustained a comminuted anteroinferior glenoid fracture without posterior dislocation or posterior involvement, despite the absence of additional trauma. This discrepancy may be explained by the arm position or the activation pattern of specific muscle groups at the time of injury. Specifically, the patient's arm was in forward flexion, adduction, and internal rotation when touching the generator. In this position, contact between the humeral head and glenoid is reduced and shifted toward the anteroinferior rim. The electric shock may therefore have reproduced a mechanism similar to anterior dislocation, leading to the observed glenoid fracture. The associated greater tuberosity fracture could also be linked to this anterior-dislocation–like mechanism, as such fractures occur in approximately 10% of shoulder dislocations (17). Nonetheless, we believe that the tuberosity fracture more likely resulted from avulsion due to sudden contraction of the infraspinatus, teres minor, and deltoid. Because the fracture was nondisplaced, no acute rotator cuff tear was observed; the partial cuff lesion detected on MRI was likely chronic. The greater tubercle fracture shows no displacement and is inherently stable. Furthermore, the rotator cuff injury is a partial tear, and the rotator cuff tendons can maintain the greater tubercle fragment in a favorable position. Therefore, under conservative immobilization of the shoulder joint, greater tubercle fractures have a high likelihood of healing (18). Since the rotator cuff injury is a partial tear rather than a “full-thickness tear,” conservative treatment is typically employed (19).

Importantly, scapular fractures caused solely by electrocution in the absence of direct trauma are exceptionally rare. Heggland et al. (20) described bilateral anterior glenoid rim fractures with anterior dislocations of both humeral heads, though that case resulted from sports trauma rather than electrical injury. Our case is consistent with earlier reports by Tarquinio et al. (7) and Kotak et al. (10), demonstrating that electrocution alone can induce shoulder fractures without concomitant falls. Interestingly, unlike the humeral head displacement commonly described by Ketenci et al. (6) in electrical injuries, our patient showed no posterior dislocation, again suggesting the role of limb positioning and muscle activation pattern during the incident.

This case also illustrates the diagnostic challenges of such injuries. Initial radiographs revealed only a nondisplaced fracture, whereas CT and MRI were required to detect the comminuted glenoid fracture and partial supraspinatus tear. This finding aligns with Beswick et al. (8), who emphasized that scapular fractures may be overlooked without high clinical suspicion and detailed imaging, underscoring the importance of advanced radiological assessment in electrical injuries.

When fractures are limited to the scapular body, conservative treatment—immobilization followed by early mobilization—is generally recommended. Surgical intervention is indicated for displaced intra-articular fractures of the glenoid, glenoid fractures associated with dislocation, coracoid fractures with acromioclavicular disruption, or fractures with neurovascular compromise (9). Most previously reported electrocution-related scapular fractures involved the body and were treated nonoperatively (Table 1). In contrast, our patient presented with a displaced comminuted glenoid fracture, which required surgery. Unlike the open reduction and screw fixation used by Heggland et al. (20), we performed arthroscopic fixation. Arthroscopy allowed direct visualization of intra-articular fragments, precise reduction, and stable fixation using suture anchors. The combination of the double-pulley and suture-bridge techniques provided multidirectional stability and minimized the risk of fragment displacement, a critical concern for long-term outcomes. Although plate-screw constructs may offer superior biomechanical strength (21), arthroscopy offers the advantage of minimal soft tissue trauma. More importantly, our patient achieved excellent clinical and radiological outcomes at 15 months, with satisfactory bone healing and restoration of glenoid morphology. These results reflect both the effectiveness of the surgical technique and the patient's adherence to rehabilitation.

Despite the favorable outcome in this case, it represents only a single report of arthroscopic management for an electrocution-induced glenoid fracture. Further studies with larger cohorts and diverse etiologies of glenoid fractures are needed to validate the efficacy and long-term benefits of this minimally invasive approach.

## Conclusion

This case likely involved an anterior shoulder dislocation caused by an electric shock injury, subsequently leading to an avulsion fracture of the anterior inferior glenoid and greater tubercle. Conservative management was applied for the greater tubercle fracture and the chronic partial rotator cuff tear, while the glenohumeral bone fragment was repositioned using arthroscopic suture anchor fixation. This approach offers a minimally invasive surgical strategy for bone and joint trauma associated with electrical injuries. The arthroscopic double pulley technique combined with suture bridge technique achieved anatomical reduction and multidirectional stabilization of the intra-articular fracture fragments. This case demonstrates that applying arthroscopic precision repair techniques to shoulder fractures caused by electrical injury can overcome the limitations of traditional conservative treatment, particularly for comminuted fractures involving articular surfaces, while avoiding large surgical incisions and extensive wound sites. Thus, arthroscopically assisted suture anchor fixation represents a novel minimally invasive treatment option for shoulder fractures resulting from electrical injuries. Further studies are needed to validate the applicability of this surgical strategy in complex intra-articular fractures caused by electrical trauma.

## Data Availability

The original contributions presented in the study are included in the article/Supplementary Material, further inquiries can be directed to the corresponding author.
